# A Critical Analysis of the British Horseracing Authority’s Review of the Use of the Whip in Horseracing

**DOI:** 10.3390/ani5010138

**Published:** 2015-03-20

**Authors:** Bidda Jones, Jed Goodfellow, James Yeates, Paul D. McGreevy

**Affiliations:** 1 RSPCA Australia, P.O. Box 265, Deakin West, ACT 2600, Australia; 2 Faculty of Veterinary Science, University of Sydney, Sydney, NSW 2006, Australia; E-Mail: paul.mcgreevy@sydney.edu.au; 3 Law School, Macquarie University, Sydney, NSW 2109, Australia; E-Mail: jed.goodfellow@mq.edu.au; 4 University of Bristol, Langford BS40 5DU, UK; 5 RSPCA, Wilberforce Way, Southwater, Sussex RH13 9RS, UK; E-Mail: james.yeates@rspca.org.uk

**Keywords:** equine, horse, pain, racing, welfare, whip

## Abstract

**Simple Summary:**

This is a critique of the British Horseracing Authority’s 2011 report, *A Review of the Use of the Whip in Horseracing*. It analyses the way the report uses science and public opinion research to reach conclusions on the animal welfare impact of whip use. Our analysis suggests that some of the report’s findings are insufficiently defended by the report and that further independent scientific review is needed to reach definitive conclusions about whip use on racehorse welfare.

**Abstract:**

There is increasing controversy about the use of the whip as a performance aid in Thoroughbred horseracing and its impact on horse welfare. This paper offers a critical analysis of the British Horseracing Authority’s (BHA) 2011 Report *Responsible Regulation: A Review of the Use of the Whip in Horseracing*. It examines the BHA’s process of consultation and use of science and public opinion research through the application of current scientific literature and legal analysis. This analysis suggests that the BHA’s findings on the welfare impact and justification for whip use are insufficiently defended by the report. These findings indicate that the report is an inadequate basis from which to draw any definitive conclusions on the impact of whips on racehorse welfare. Further review is needed, undertaken by an independent scientific body, to advance this debate.

## 1. Introduction

In 2011, the British Horseracing Authority (BHA) reviewed its rules for whip use. Prior to this review, the BHA stated that it had the world’s most exhaustive instructions on the use of the whip in racing [1]. Nevertheless, during the lead up to the review, two incidents of excessive whip use by winning jockeys brought whip use under public and media scrutiny, including prompting one Member of Parliament to write to the BHA about whip overuse [2].

The terms of reference for the review were purposely drafted in broad terms: “To review the use of the whip in Horseracing in Great Britain”. This provided the BHA with sufficient flexibility to consider not only the adequacy of the rules themselves but also the broader appropriateness of whipping from an ethical, animal welfare and public perspective. The review was conducted by an internal BHA committee (the review group), which finalised its inquiry with the publication of the Report, *Responsible Regulation: A Review of the Use of the Whip in Horseracing*, in September 2011 [3].

The Report itself was not presented as a “scientific”, peer-reviewed paper, but it did include some scientific references and included a large amount of statistics and biological terms alongside its arguments. The goal of the current article is to evaluate the evidence presented in the report through a comparison to the available science and, where specific data are lacking, on common sense assumptions based on scientific animal welfare principles (e.g., learning theory).

It considers each section separately, identifying salient issues that might be interpreted differently. This evaluation suggests that the Report suffers from a number of deficiencies, including its failure to provide adequate scientific support for its final recommendations and flaws in the review’s consultation process, public survey research and data analysis. As such, the Report arguably fails to provide an adequate justification for the continued use of the whip in horseracing.

## 2. Analysis of the Report’s Findings and Recommendations

The Report is organised into seven chapters, which take the reader through the review process; this section of the paper is similarly organised using the same headings as in the Report. Where reference is made to a specific section of the Report, the paragraph number is provided in brackets, e.g., (1.23).

### 2.1. The BHA’s Approach to Animal Welfare and the Whip

The Report begins its first section by asserting that, as the regulator for horseracing in the UK, the BHA takes its responsibilities seriously and that safeguarding the welfare of racehorses is a priority. The starting point for the Review itself is that “the use of the whip should only continue if it was considered *necessary* and *appropriate*” (1.10) (emphasis added).

Firstly, it is important to establish the meaning of the term *necessary*. This concept has been given considerable thought by the English Judiciary in the context of applying the prohibition on causing an animal *unnecessary* suffering under animal welfare legislation [4]. The leading case on this question is *Ford v. Wiley* [5], an old English High Court decision from 1889. *Ford v. Wiley* developed a test to determine when suffering caused to an animal could be deemed to be unnecessary in a particular set of circumstances. The *Ford v. Wiley* test requires consideration of both the *object* of the practice under question, and the *means* employed to achieve that object. The court must first consider whether the object sought to be achieved was legitimate. Also implicit in any necessity assessment is the notion of causality. For the relationship between means and object to be considered necessary, there must be a causal connection between the two. In other words, if the means do not serve to fulfil the intended object, they cannot be described as proportional. Indeed, pain-causing means in the absence of a connection with a legitimate object will be deemed “unnecessary” as such means will be considered to have no corresponding purpose. Whether something is “necessary” also requires a consideration of less harmful alternatives: if there are other less intrusive means by which the object can be attained, they should be adopted. This requirement is also reflected in Section 4(3)(a) of the *Animal Welfare Act 2006* (UK), which states that in determining whether the suffering is unnecessary, the court should consider “whether the suffering could reasonably have been avoided or reduced”.

Thus to determine whether whip use *is* necessary, it is appropriate for the review to consider what the appropriate purposes are for which whips might be acceptably used. The Report sets out three purposes, described in the BHA *Guide to Procedures and Penalties* [6] as “safety”, “correction” and “encouragement”, highlighting also that the Guide states that the whip “should never be used to coerce”.

The Report states that the use of whips for safety is acceptable to all those consulted, and goes on to conclude that whip use is “a necessary aid to horsemanship”, (1.11; 2.25). The first point is to be expected, and seems sensible: safety (in particular to avoid major crashes) seems a convincing rationale for the relatively lower harm of a single whip strike. However, the argument that whip use should be acceptable where it is necessary for safety is not the same as the question of *whether* whip use is necessary for safety or *when* it is so necessary. The use of whips for correction is also explained in terms of safety (indeed the Report regards the term correction as “superfluous”; 1.23). Three examples of using the whip for safety are given: where a horse may (a) “shift ground”, (*i.e.*, deviate from a correct path); (b) need rebalancing; or (c) “back off a jump”. In the first instance of shifting ground, the Report suggests that the reins should be used prior to whip use to realign the horse (1.17). Using the whip to maintain balance is not discussed at length (although a later section highlights that jockeys stated that restricting the use of the whip to the backhand manner only would have an adverse effect on their ability to assist the horse in maintaining its balance, particularly in the later stages of a race; 2.29), but it is not made clear how being whipped can improve a horse’s proprioceptive reflexes. Using the whip to make a horse go over a jump may presumably increase safety if it is necessary to prevent the horse injuring itself or others. However, a plausible explanation for a horse backing off a jump is that it has an adaptive motivation not to jump it, for example due to fatigue. In such cases, whipping a horse may lead to mistakes over fences they would otherwise have refused to jump, potential injury to that horse or others, and could plausibly be described as coercion. Thus preventing a horse backing off is not *always* necessarily a matter of increasing safety: the Report does not provide sufficient evidence that hitting a horse before a jump does increase safety.

In particular, it remains an open question *when* the use of the whip can be expected to genuinely increase safety and whether restricting whip use would act to reduce or increase safety. To help consider this, it would have been instructive for the Report to have sought information on the impact of the Norwegian whip rules on jockey safety. Norway has strictly limited whip use since 1982, including removing whips completely from most flat races since 2009, and while the industry is comparatively small, there is no evidence that the safety of jockeys and horses has been negatively affected [7].

The third purpose is encouragement, which is variously explained as focusing and concentrating a horse so that it performs at its best during a race and stimulating the horse to realise its potential in the race. In other words, the whip is used to make the horse go faster. The Report states that a “common misconception is that the main reason jockeys use the whip is simply to make the horse go faster” (1.21), but it is difficult to see what else “encouragement to give its best” can mean insofar as the superlative “best” implies encouraging the horse to the limit of its capacity. The Report rightly considers that hitting horses that are “out of contention” is unacceptable. However, out of contention is not defined. Given that 98% of horses were whipped in the Evans and McGreevy study of whip use [8], it seems that the circumstances when horses are considered to be out of contention by the racecourse stewards may be limited.

### 2.2. How the Whip is Currently Used in Horseracing

The second section begins by considering whip offences (*i.e.*, whip use that breaches the rules [9]: see Table 1). It does not take into account how the whip is used according to the rules, which would have been a useful analysis. More offences were seen when the going was slow (2.8), there was a winning chance (2.9), a close finish (2.10) and with higher-quality races (2.11). Nearly two-thirds (64%) of whip-use offences occurred when a horse had a winning chance, and whip offences are more likely in higher-grade races. This suggests that illicit whip use is used for coercion rather than safety, although it does not tell us anything about allowed whip use.

It then proceeds to set out a novel semantic distinction between the terms issue and problem, and then records that while those consulted by the review considered whip use to have the potential to be a welfare issue, they did not view it as a welfare problem when padded whips are used under appropriate controls (2.21), a view supported by the Report (2.22). However, rather than making this distinction, a more useful differentiation might have been between justified use of the whip and wrongful use of the whip. Even if striking a horse with a whip *will* (or will have the potential to) compromise its welfare—the question is how much and whether it is justified. By virtue of its inherently noxious nature, whip use in racing is an “issue”; furthermore it was, and arguably remains, a “problem” that requires resolution and change—which we assume was the purpose of the Review.

The distinction between “issue” and “problem” was, at best, confusing and potentially misleading. Such definitional techniques lead to inconsistencies in the consultation group’s responses. For example, the Report notes that: “Those consulted by the Review Group expressed the view that the current use of the whip in Racing is not a welfare problem … due to the design of the only whips approved by the Authority for use in races in Great Britain, and the Authority having in place appropriate controls on their use.” This conflicts with the view that the majority of those consulted were in favour of additional restrictions on whip use (2.27). These apparently contradictory statements may have been made because jockeys (and others) are placed in an undesirable quandary—they must not use the whip “excessively” but they must also “ride the horse out”, and so feel that additional rules around whip use would help provide further guidance on achieving this balance.

**Table 1:** Verbatim instructions on acceptable and unacceptable use of the whip in the BHA rules (BHA, 2008).

Acceptable Use of the Whip	Unacceptable
Showing the horse the whip and giving it time to respond before using it	Hitting: To the extent of injury With the whip arm above shoulder height Rapidly without regard for their (the horse’s) stride With excessive force With excessive frequency Without giving the horse time to respond
Using the whip in the backhand * position for a reminder	Hitting horses that are: Showing no response Out of contention Clearly winning Past the winning post
Having used the whip, giving the horse a chance to respond to it before using it again	Hitting horses in any place except: On the quarters (hindquarters) with the whip in either the backhand or forehand position Down the shoulder with the whip in the backhand position; unless exceptional circumstances prevail
Keeping both hands on the reins when using the whip down the shoulder in the backhand position	
Using the whip in rhythm with the horse’s stride and close to its side	
Swinging the whip or actually using it to keep the horse straight	

* Backhand position means the whip is held as one would hold a ski-pole.

### 2.3. The Scientific Evidence Base

The Report’s assessment of the available literature rightly highlights that there is very little science on any aspect of whip use. Only 11 scientific papers are cited: two of these are human-focused studies and only four actually focus on whip use in racing. The Report identifies that there are no studies that objectively and directly assess horse welfare in relation to the use of the whip in races (3.5). There is no science to tell us about how to design the whip, where it is best used on the horse, the force to be applied, how often it should be used, the effect it has locally, and if, or how, pain is experienced by the horse.

This overall lack of evidence *per se* is therefore not a criticism of the Report. What is challengeable is that, despite this vacuum of evidence, the Report takes the opportunity to make categorical statements. For example, while highlighting the risks of improper whipping, they assert that “controlled use of a specific whip does not cause pain” (Box 1, p. 16) and that “current scientific evidence broadly supports the continued use of the whip in Racing” (3.35). The Report is here trying to highlight that improper whipping may be painful, but that “proper” whipping is not, but on no basis.

#### 2.3.1. Clinical Assessment

At the racetrack, horses are examined by a BHA Veterinary Officer for the impact of whip use only when “any whip use that might place the horse’s welfare at risk is identified”. The key clinical sign they are looking for is the presences of weals. The Report states that only around 20 episodes of weals were seen per year, and while in such cases Veterinary Officers looked for associated signs of inflammation, discomfort or pain, none were observed (3.7). One candidate explanation for this zero prevalence is that the protocol is inadequate to determine whether pain has been caused. This may, in part, be due to the lack of robust scientifically evaluated methods to quantify the effects of whip strikes, and highlights the need for further research in this area. In terms of the Report, this limitation means that it is misleading to conclude that there is no pain from whip strikes (or that this finding has any significant implications as to whether there is pain). The absence of evidence should not be taken, or presented, as evidence of an absence of pain.

#### 2.3.2. Physiological Aspects

The Report argues that, using the analogy of holding a hot cup of coffee, the experience of a horse in response to being struck by the whip is on a continuum from pleasure to pain (3.13) and that only when things get really bad would the effect of a whip be perceived as painful. This comparison is irrelevant or misleading. There are various critical differences between thermal pain *versus* mechanical pain and between the reasoning ability of humans *versus* horses. It is unclear how the first phase of this continuum—causing pleasure or only mild discomfort—applies to whip use. Holding a cup of hot coffee is also a voluntary action, which the human controls; when the subject is in control of the aversive stimuli, welfare is much less likely to be compromised [10]. In racing, however, the horse has no control over whether, when and with how much force it is whipped.

The Report also suggests that horses may experience “sportsman’s analgesia” where the appreciation of pain is altered by the physiological effects of peak exercise, but there is no empirical evidence from studies on horses to support this view. Indeed, the author cited [11] identifies that only a limited amount of research has been conducted in this area. Recent research suggests that pain perception may be enhanced, rather than reduced, in some stress-related states [12,13]. And if exercise does induce an analgesic state, this could actually imply that horses would need to be struck especially hard to overcome this. The prospect of pain from post-whipping inflammation once the adrenaline response has subsided is not considered.

That exercise may reduce pain perception is only put forward as a suggestion in the Report itself, but in an opinion piece written at the time of its public launch, Dr. Tim Morris, BHA Director of Equine Science and Welfare, treats this suggestion as though it were a key finding: “The BHA also looked very closely at the animal welfare science behind the effects of the whip on horses in the specific context (and this is important) of adrenaline-fuelled race conditions. What we *found* was that under such conditions, when a horse is in a state of high physiological and mental excitement, the use of an energy-absorbing whip does not cause pain if used within strict limits. In sports science this is often termed ‘sportsman’s analgesia’, and it means that whilst the whip stimulates a horse during a race, it won’t cause pain or suffering if used properly.” ([14], emphasis added).

The Report does not in fact present any evidence to support the statement that the whip does not cause pain to racehorses.

#### 2.3.3. Behavioural Studies

The Report states that the use of behavioural techniques and assessment is important, yet they have not been applied to the use of whips in race conditions. Despite it being a claimed priority for the industry, there appears to have been no scientific research funded to investigate the welfare impacts of whip use, which might be expected to have been a priority for research funding. Due to this lack of available evidence, the Report’s commentary under this heading relies heavily on “general observations” from “those involved in racing” rather than objective information.

The Report does not provide any detailed explanation of the way in which whips are supposed to alter horses’ behaviour, in particular with regard to ‘encouraging’ them. The Report’s initial phrasing implies that a horse has an innate desire to “perform at its best” and “realise its potential”, which the whip use “enables” (1.20; 1.22), but there is no evidence that the horse has any concept of “its potential”. Nor does it seem plausible that an external stimulus (the whip) would increase a horse’s focus on an unassociated stimulus (the jump). Such additional stimuli would more likely be a distraction, according to mainstream concepts of attention (e.g., gate theory). An alternative motivation might be of positive reward. Indeed, the terms “encouraged”, and to a lesser extent “motivated”, imply an entirely positive experience. However, conditioning theory would suggest that whip use can provide a reward only (a) as a conditioned stimulus associated with a pleasant unconditioned stimulus, which seems unlikely on mainstream training regimes; or (b) as an unconditioned stimulus used within a mechanism of negative reinforcement. The latter relies on the whip use itself being aversive.

Two hypotheses suggested in the Report (3.19) are that the whip is used as negative reinforcement (without being rewarding) or as punishment (e.g., for slowing down, shifting ground or backing off a jump). Another suggested hypothesis is that the whip is a conditioned stimulus associated with other conditioned stimuli (e.g., other cues; 3.20). Again, the most plausible explanations of a whip’s effectiveness in prompting a horse’s response (*i.e.*, moving away from the impact of the whip) rely on the whip, or associated stimuli, being aversive. It is difficult to see what other mechanism can account for the whip’s role in steering, and these remain the most parsimonious explanations of the effectiveness of whips as “encouragement”.

The Report fails to explain the whip’s effectiveness, yet asserts that the most intuitively plausible explanation—that they are aversive—is false. In particular, it states that in the context of a race, a horse can be encouraged and motivated without in any way being abused or in pain (1.20) and that “encouragement may mean an increase in speed but only in certain circumstances in which the whip is used in a way so as not to cause pain” (1.22), and that “the general observation of those involved in racing is that, with very few exceptions, horses, both during a race and at other times, do not display overtly aversive behaviour towards whips used in racing” (3.18). In fact, it is difficult to see how else whips could motivate behaviour.

In recognition of the possible mechanism of negative reinforcement, the Report places a number of conditions on what it deems to be acceptable use of the whip (Box on p. 12), in addition to those in Table 1, and that “The whip may only be used on the horse’s body where, in the context of the race, it will not cause pain”, limiting the number of uses and requiring the “specifically designed energy-absorbing whip that does not cause pain when properly used”. These conditions could be argued to be consistent with the argument that the energy-absorbing whip does not cause pain when used a limited amount on certain parts of the body. They are beneficial suggestions, however, they leave open relevant questions about what the mechanism for the padded whip’s effect is. Given the existence of a plausible mechanism—negative reinforcement—the burden of proof would logically fall upon the Report to substantiate the claim by explaining the mechanism and/or providing supporting evidence.

#### 2.3.4. Effects on Safety

A similar problem arises when the Report discusses the relationship between whip use and safety. One study reported identified whip use as a potential risk factor for horse falls [15], and specifically suggested that there were increased risks of falling from whip use “immediately before an obstacle is jumped and whilst a horse is progressing in position during the race. This may be due to increased speed and/or the horse being unbalanced by whip use.” A second study cited, which used analysis of racetrack patrol videos, suggested that 38% of breakdown injuries occurred after whip use [16]. Possible explanations for this association include that riders respond to horses beginning to pull up with lameness by using the whip, which increased speed from whip use reduces safety or that riders use the whip to avert a potential fall as a breakdown begins (*i.e*., for genuine safety). These findings may suggest that whip use actually decreases safety, and that whip use for correction may be counter-productive. No evidence is presented to evaluate the use of the whip for safety unassociated with injury. The conclusions in this section of the Report are instead based on jockeys’ opinions and jockeys have stated that they *must* carry a whip for safety purposes (3.26).

#### 2.3.5. Effects on Performance

The Report criticises the methodology of the only study [8] that has questioned the effect of whip use on performance. It argues that “The applicability of this study to the British situation may be limited because it was in Australian flat races where unlimited frequency of whip use is allowed in the final 100 m”, despite the fact that this could, intuitively, lead to a greater effect (*i.e*., if no increase in velocity were seen with higher whip use, then this would be expected to hold for less whip use). The Report adds that “the study has been criticised scientifically for its small sample size” despite its results being statistically valid. The study’s authors have responded to online criticism and there has been no peer-reviewed scientific objection to it. The Report fails to acknowledge that this study found that, on average, whip use was not related to performance, challenging the necessity and efficacy of whip use at the end of a race.

### 2.4. The Whip: Energy-Absorbing Design

The Mahon-designed whip is correctly described as energy-absorbing. A comparative study conducted on a range of whips found that when the same force was applied, the end of the padded whip left a shallower depression in a slab of Plasticine than other traditional whip designs [17]. At the time of the review, this was, to the authors’ knowledge, the only published empirical study of the impact of the padded whip, although it is not cited in the Report. There is no research to indicate that this whip is pain-free, although it is reasonable to assume that, if it were used in the same way and with the same force as a traditional whip, it would have less impact, thereby causing less tissue damage and thus less response with less-intense associated pain in general terms. However, still photographs and high-speed video footage of whip use during Australian flat races indicate that when padded whips are used, both the padded and unpadded sections can come into contact with the horse [18]. Recent research (since the review) suggests that strikes where the unpadded section of the whip makes contact with the horse are more common than when only the padded section makes contact [18]. Additionally, there is recent evidence (since the review) that backhand strikes land with more force than forehand strikes [19].

### 2.5. Public Opinion Research

The Report presents a summary of the findings of a public-opinion survey, the key finding of which was that 57% of respondents said they strongly or somewhat strongly agreed that whip use should be banned completely. The Report then goes on to highlight that this view was held predominantly by women (5.8). The Report’s analysis of the public survey data appeared to discount the 57% of views of those expressing concerns about whipping, insofar as the Report then recommended the development of a communications strategy to “maximise understanding” of the broader community about the BHA’s views on whipping (5.31 and 5.33).

Respondents were then provided with an “explanation” of whip use and the “pain free nature of its design” (5.11) before being asked the same question again:

*Question 13: “As stated previously, the whip used in racing in Britain is a cushioned whip and has been specifically* *designed not to cause pain …”*

(p69)

To state that the whip has been designed not to cause pain risks respondents inferring that it does not cause pain. After this explanation, fewer than half the respondents actually changed their minds. This suggests either that respondents did not believe the implication that the cushioned whip did not cause pain or that they had more deeply held views that whipping horses should not be allowed. The Report suggested from this that there was a lack of understanding in the wider community of the industry’s arguments for why whips are needed and the effect of the whip on horses. This finding is not surprising given that, despite the longevity and prevalence of whipping in racing, the answers to these questions are not yet fully understood.

In addition, the survey found that 74% of people with an interest in horseracing and betting (*i.e*., those who had placed a bet within the past 12 months) would not change their betting habits if the use of whips was banned completely. An additional 12% stated they would actually be “much more” or “somewhat more” likely to bet, while only 8% said they would be less likely.

### 2.6. The Penalty System

The Report proceeds to several recommendations to decrease the use of whips in UK horseracing. In particular, the Report recommended that the maximum acceptable number of whip strikes in flat races be reduced from 15 to 7, with a maximum of 5 strikes in the last furlong. In jump races the maximum number of strikes was reduced from 16 to 8, again with a maximum of 5 strikes after the last obstacle. The bases for these numbers were not made clear, and the Report acknowledged them to be “arbitrary” (6.4).

The Report places considerable emphasis on the effectiveness of current arrangements in enabling stewards to enforce the new rules and on more stringent penalties acting as a deterrent to breaching the rules. Counting whip strikes may be difficult and requires review of the action of individual jockeys from video footage of races. Accurately assessing the whipping actions of individual jockeys from head-on cameras and making a visual appraisal of the amount of force being used are difficult tasks, particularly when views are obscured by other horses and riders. A head-on view also does not help the stewards determine where, on the horse’s anatomy, the whip has struck. These limitations might be improved by the development of aids to enforcement, such as multiple cameras or slow-motion cameras, and the Report’s conclusions suggest that these should be developed and adopted.

Racing stewards’ judgments are difficult and subjective. They are blurred further by the stewards’ need to estimate and then factor in the degree of force and frequency of use [1]. Furthermore, the BHA’s disciplinary enquiries usually relate to how the horse ran, for example, a failure to run a horse on its merits; interference with other runners; excessive use of the whip. The first and last of these imply apparently contradictory concerns insofar as evidence that a jockey has run a horse on its merits (*i.e.*, ridden a horse out) is provided by, and may require, his use of the whip. Instead, jockeys may be accused of hitting the horse on multiple occasions without giving it time to respond. This point is critical because the response of the horse to the whip cannot be assumed. In other words, to some extent the use of the whip, if only as a test of whether the horse has the energy to respond, becomes obligatory. Some horses may speed up; some may move laterally; others may slow down.

### 2.7. Jockey Training

The Report finishes with a very welcome and appropriate discussion of jockey training, which complements the previous discussions.

## 3. Conclusions

The undertaking of the BHA review was, in the opinion of the present authors, an excellent and well-timed opportunity to examine the use of the whip in horseracing in relation to horse welfare. Indeed, many of the recommendations (in particular those not mentioned in this paper) were welcomed by many involved in racing and welfare organisations. However, the Report also concluded that whip use is not painful, that whip use is not a welfare problem, and that whip use is necessary for safety and encouragement, without adequate evidence to support these claims. This highlights some more general concerns about the review process.

First, the review was undertaken by the BHA, an organisation that exists to promote, as well as regulate, the racing industry [20]. As the use of horses within the industry often conflicts with horse welfare [21], this gives rise to the risk of a conflict of interest, or at least of the perception of such a conflict, for the BHA in carrying out the review. The tenor of some of its conclusions suggests that this conflict affected the process and outcomes of the Report. This concern particularly relates to issues on which the Report fails to provide an adequate justification for the continued use of the whip in horseracing. In the absence of an independent review of the use of the whip, the BHA’s review is welcome, but governments should be cautious when depending on racing industries alone for advice on horse welfare.

Second, the Report repeatedly highlights the “extensive consultation” undertaken throughout the review process, yet of the 16 groups consulted, only three have no direct association with the racing industry. The review group did not report any consultation with the International Society for Equitation Science (ISES) or with any equine veterinarians who do not have a direct connection with racing. It is also unfortunate that Animal Aid felt it necessary to withdraw from the process. The omission of these groups from the consultation process is significant, as Animal Aid advocates a ban on whip use and ISES has recently published a position statement that actively discourages the use of whips in horseracing [22].

Third, the question arises whether the Report’s conclusions were genuinely intended to be implemented. The BHA backed down from a number of its key recommendations within days of the amended rules being announced. Following pressure from the Professional Jockeys Association, the BHA removed the numerical limits on the number of whip strikes in the final stages of a race, removed jockeys’ riding fees from any penalty, and increased the threshold before any prize money could be forfeited from jockeys who had been suspended as a result of breaching the rules [23]. Then, only a month after the original rules were put in place, these penalties were relaxed even further by reducing the five-day ban for one whip strike over the maximum to two days, and allowing the Stewards to exercise discretion in determining whether breaches have occurred [24]. Three months later, the rules were further diluted, removing automatic breaches for whip use above a set maximum number of strikes leaving referral of each incident to the discretion of the Stewards [25]. Thus the use of the whip as now permitted by the BHA no longer even reflects the objectives of its review.

Accordingly, the Report should not be taken as representing an authoritative evaluation of the use of whips in racing, but simply the formal position of the British racing industry as presented by the BHA, which represents the industry’s interests. As such, the Report should not be used by government as the basis for public policy on the use of whips. Where such interests conflict with a matter of broad public concern, such as horse welfare, government has a responsibility to ensure that resolutions to such conflict are governed via fair and democratic processes. Routinely deferring legitimate public concerns and questions about racehorse welfare to the BHA (e.g., [26]) fails to achieve this and will affect community confidence in governance arrangements for racehorse welfare.

Nevertheless, the BHA review can be seen as the start of the process of reviewing the use of whips in horseracing—a use that has been largely taken for granted in many countries. Further research is demonstrably needed to investigate the issues central to continued defence of whip use, in particular its usefulness in safety and its mechanism of action. Further ethical analysis is needed to consider the legitimacy of encouragement as a value. Further public consultations are also needed to determine whether opposition to whip use is based on sound understanding. Finally, a further review should be undertaken by an independent scientific body to avoid conflicts of interest and provide an objective evaluation of the available evidence on the impact of whip use on racehorse welfare.
